# Reliability and Validity of Facial Check Sheet (FCS): Checklist for Self-Satisfaction with Cosmetic Acupuncture

**DOI:** 10.3390/medicines8040018

**Published:** 2021-04-11

**Authors:** Hiroshi Kuge, Hidetoshi Mori, Tim Hideaki Tanaka, Ryouta Tsuji

**Affiliations:** 1 Department of Anesthesiology Pain Clinic, Osaka Medical and Pharmaceutical University Hospital, Osaka 569-8686, Japan; sherry@kha.biglobe.ne.jp; 2 Department of Health, Faculty of Health Science, National University Corporation Tsukuba University of Technology, Tsukuba 305-8521, Japan; h.mori.tsukuba@gmail.com; 3 Information Science Center for Humanities and Sciences, School of Health Sciences, Ibaraki Prefectural University of Health Sciences, Ibaraki 300-0394, Japan; 4 Department of Acupuncture, Faculty of Health Sciences, Morinomiya University of Medical Sciences, Osaka 559-8611, Japan; tsuji@morinomiya-u.ac.jp

**Keywords:** acupuncture, cosmetic technique, face, skin wrinkling, reliability, validity, questionnaires, survey methods

## Abstract

**Background:** In recent years, cosmetic acupuncture has gained popularity among individuals interested in improving their facial appearance. We have created an original facial check sheet (FCS) to obtain cosmetic acupuncture patients’ perspectives on treatment outcomes. This study examined the reliability and validity of FCS. **Methods:** We conducted an Internet survey on the appearance of the facial region among Japanese women. A reliability analysis was performed between each item of FCS. A multiple comparison procedure was used to determine the relationship between the age group, the FCS score, and the number of terms used in the open-ended question. **Results:** The most frequently stated concern was blotchiness and hyperpigmented spots (47.2%, n = 67). The FCS items showed reliability (Cronbach α = 0.871). The number of extracted terms and the FCS score showed a moderate correlation (r = 0.407; *p* < 0.001). There was a significant relationship between age and FCS score (*p* = 0.005, r^2^ = 0.255), which indicated that the FCS score increases with aging. **Conclusions:** The FCS can be used as a practical tool to evaluate facial appearances and assess satisfaction levels of patients who underwent cosmetic acupuncture or other facial skin rejuvenation procedures.

## 1. Introduction

Acupuncture has been practiced for many centuries in China, Japan, and other Asian countries. It is one of the primary therapeutic modalities of traditional East Asian medicine that has been used to treat a wide variety of health conditions [1]. In recent years, acupuncture has gained popularity among individuals interested in improving their facial appearance [2,3,4]. Applications of acupuncture intended for antiaging and skin rejuvenation benefits have been prompted in various terms such as “facial revitalization acupuncture”, “face-lifting acupuncture”, or “cosmetic acupuncture”.

In educational training settings, growing numbers of acupuncture schools have begun to incorporate cosmetic acupuncture in their extracurricular courses. Some esthetic salons have been utilizing cosmetic acupuncture in an attempt to increase facial muscle tone. Previous reports suggested that cosmetic acupuncture in conjunction with standard facial care may induce desired facial skin-tightening effects, possibly from synergistic effects of treatments [5,6,7]. However, there is insufficient evidence that supports the efficacy and safety of cosmetic acupuncture [8].

Various assessment methods have been used to evaluate the effectiveness of cosmetic acupuncture. Previous studies have used objective indexes, such as facial skin temperature, blood flow, dermal fluid, and oil content [2,5]. In personal care settings, the treatment outcomes have mainly relied on subjective observation by clients or practitioners.

For the subjective analysis of cosmetic acupuncture, various survey methodologies have been utilized, including the visual analog scale (VAS), ordinal scale, and questionnaires. As an informative tool for quantifying skin aging, Guinot et al. created an aging skin score [9]. Klassen et al. developed an adverse effect checklist for minimally invasive cosmetic procedures [10]. However, these questionnaires do not indicate the degree of self-satisfaction following the treatment.

Thus, we created an original facial check sheet (FCS) to obtain cosmetic acupuncture patients’ perspectives on treatment outcomes. The FCS consists of 12 checklist items. The FCS items were scored by the ordinal scale. Although the testing sample was small, we were able to confirm the reliability of the FCS items [11]. 

Although the previous studies demonstrated the changes in the FCS scores after the application of acupuncture in the facial region [5,6,7], the relationship between the FCS items and self-observatory facial status has not been studied. As well, the validity of the FCS has not been tested. In this study, we examined the reliability and validity of the FCS that can be incorporated into the outcome assessments of cosmetic acupuncture.

## 2. Materials and Methods

### 2.1. Survey

In this cross-sectional study, we conducted an Internet survey on the facial region’s appearance among Japanese women. This study was conducted from July 2017 to September 2017.

### 2.2. Participants

The eligibility criteria for the subjects were Japanese females who were residing in Japan. Males, as well as the respondents who did not state their age, were excluded. Among 204 respondents who consented to this study, 191 respondents (93.6%) were eligible. They were between 26 and 72 years old (SD: 7.4).

### 2.3. Ethical Considerations

All study participants signed informed consent before enrollment in the study.

This study was approved by the Ethics Committee of Tsukuba University of Technology (approval number: H29-7) and registered in the UMIN Clinical Trials Registry (UMIN000041417).

### 2.4. Questionnaires

The questionnaires of the Internet survey in the present study were based on the FCS that we originally created [5,6,7]. The survey consisted of closed and open-ended questions.

#### 2.4.1. Presence/Absence of Dermatological Conditions (Single Choice)

(1) Currently under clinical care, (2) Previously under clinical care, (3) Treated with over-the-counter drugs, and (4) No history of dermatological conditions.

#### 2.4.2. Facial Care Status (Multiple Choice)

(1) I do skincare by myself more than once a week, (2) I receive professional skincare treatment(s) more than once a month, (3) I receive facial cosmetic acupuncture treatment(s) more than once a month, (4) I receive general (systemic) acupuncture treatments(s) more than once a month, (5) I only apply makeup before going out (no skincare treatment), and (6) I do not apply anything to my face.

#### 2.4.3. Self-Evaluation of Facial Appearance

(1) Periorbital lines (crow’s feet), (2) Glabellar frown lines (forehead furrows), (3) Horizontal forehead lines, (4) Nasolabial folds (smile lines), (5) Infraorbital folds (mid-cheek lines), (6) Marionette lines (mandibular folds), (7) Infraorbital skin laxity, (8) Cheek laxity, (9) Corner of the mouth lines/laxity, (10) Facial shape/contour, (11) Complexion, and (12) Blotchiness and hyperpigmented spots (Table 1).

#### 2.4.4. The Level of Concern Regarding Each Item Was Rated by the 5-Point Likert Scale

(1) Not at all concerned, (2) Slightly concerned, (3) Somewhat concerned, (4) Moderately concerned, (5) Extremely concerned, and (0) I do not understand the term.

The respondents were allowed to choose one answer per question. The response (0) “I do not understand the term” was treated as a missing value for the analysis.

#### 2.4.5. Open-Ended Questions

The participants were asked to describe the area(s) of concern(s) of their face and state their age.

### 2.5. Sample Size

One hundred forty-two samples were required based on 15 parameters, effect size r^2^ = 0.13, the type I error (0.05), and the type II error (0.8). R Ver.3.6.1 was used for the sample size calculation.

### 2.6. Statistical Analysis

Among 191 eligible respondents, 142 samples were randomly selected for the analysis. 

A normal distribution of age was tested by the Shapiro–Wilk normality test. Each item in the closed-ended questions was aggregated. Reliability analysis was performed between each item of the FCS.

Terms were extracted for coding from the open-ended question box where the respondents freely stated area(s) of concern(s) of their face. Spearman’s rank correlation coefficient was used to analyze the relationship between the FCS score and the terms in the statements. A multiple comparison procedure was used to determine the relationship between the age group, the FCS score, and the number of terms used in the open-ended question. Furthermore, three parameters of age, the FCS score, and the number of terms were examined by multiple regression analysis (direct injection method).

All data were analyzed using the Statistical Package for the Social Sciences (SPSS software Version: 22, NY, USA). P-values less than 0.05 were considered significant.

## 3. Results

Among the 142 random respondents, their ages ranged from 26 to 72, with an average age of 44.3 (SD: 7.5). The age distributions were as follows: 20–29 (0.7%, n = 1), 30–39 (28.2%, n = 40), 40–49 (49.3%, n = 70), 50–59 (19.7%, n = 28), 60–69 (1.4%, n = 2), and 70–79 years old (0.7%, n = 1). Age was not normally distributed (*p* = 0.020).

### 3.1. Aggregate Count of Each Item

#### 3.1.1. Presence/Absence of Dermatological Conditions (Single Choice)

A total of 84 (59.2%) of respondents had no history of dermatological conditions, whereas less than 10% of respondents were receiving clinical dermatological care.

#### 3.1.2. Facial Care Status (Multiple Choice)

A total of 42.3% (n = 60) of respondents selected (5) I only apply makeup before going out (no skincare treatment), whereas 10% of respondents were receiving acupuncture or esthetic treatments.

### 3.2. Open-Ended Question

Terms were extracted for coding from the open-ended question box where the respondents freely stated their concerns regarding their face. Table 2 shows the 33 terms that were extracted from the open-ended question statements. On average, 2.7 extracted terms were used per respondent (ranging from 0 to 6 terms). The most frequently stated concerns were blotchiness and hyperpigmented spots (47.2%, n = 67).

### 3.3. FCS Items and Reliability

The FCS items showed reliability (Cronbach α = 0.871). The FCS item (0) “I do not understand the term” was treated as a missing value. The response was distributed as follows: infraorbital folds (59.2%, n = 84), marionette lines (61.3%, n = 87), glabellar frown lines (0.7%, n = 1), nasolabial folds (0.7%, n = 1), and facial shape/contour (0.7%, n = 1).

#### 3.3.1. Relationship between FCS Items and the 33 Terms That Were Extracted from Freely Stated Area(s) of Concern(s) of Their Face

The correlation between the term “wrinkle” and the FCS items were as follows: periorbital lines (r = 0.425; moderate, *p* < 0.001), horizontal forehead lines (r = 0.192; very weak, *p* = 0.022), glabellar frown lines (r = 0.273; weak, *p* = 0.001), nasolabial folds (r = 0.188; very weak, *p* = 0.025), infraorbital skin laxity (r = 0.240; weak, *p* = 0.004), cheek laxity (r = 0.170; very weak, *p* = 0.043), and corner of mouth lines/laxity (r = 0.175; very weak, *p* = 0.037).

The correlation between the term “skin laxity” and the FCS items were as follows: periorbital lines (r = 0.194; very weak, *p* = 0.021), nasolabial folds (r = 0.225; weak, *p* = 0.007), infraorbital skin laxity (r = 0.281; weak, *p* = 0.001), cheek laxity (r = 0.314; weak, *p* < 0.001), corner of mouth lines/laxity, (r = 0.245; weak, *p* = 0.003), and facial shape/contour (r = 0.252; weak, *p* = 0.002).

The correlation between the term “nasolabial folds” and the FCS items was as follows: nasolabial folds (r = 0.444; moderate, *p* < 0.001) and cheek laxity (r = 0.182; very weak, *p* = 0.030).

The correlation between the term “dull skin” and the FCS item “complexion” was weak (r = 0.221, *p* = 0.008).

The correlation between the term “facial pores” and the FCS item “complexion” was weak (r = 0.265, *p* = 0.001).

The correlation between the term “blotchiness and hyperpigmented spots” and the FCS item “blotchiness and hyperpigmented spots” was weak (r = 0.378, *p* < 0.001).

The correlation between the term “corner of the eye” and the FCS items was as follows: periorbital lines (r = 0.264; weak, *p* = 0.002) and horizontal forehead lines (r = 0.188; very weak, *p* = 0.025).

The correlation between the term “forehead” and the FCS item “horizontal forehead lines” was weak (r = 0.206, *p* = 0.014).

The correlation between the term “dry skin” and the following FCS items was very weak: “dry skin” (r = −0.117–0.091, *p* = 0.166– 0.863), “resilience of the skin” (r = −0.068–0.148, *p* = 0.078–0.936), and “dark circles around and under the eyes” (r = −0.136–0.093, *p* = 0.107–0.916).

The number of the extracted terms and the FCS score showed a moderate correlation (r = 0.407; *p* < 0.001).

#### 3.3.2. Relationship between Age Groups, FCS Score, and the Number of Extracted Terms

Regarding the FCS score, there was a difference between ages 30 and 39 (2.4 points, SD: 0.8) and the age of 50 to 59 (3.1 points, SD: 0.9) (*p* = 0.002). 

A score of 2.8 (SD: 0.9) between the ages of 40 and 49 was not different between the ages of 30 and 39, and 50 and 59.

In regards to the extracted terms, there was no difference between the age groups: 30 to 39 years old (2.0 terms, SD: 1.3), 40 to 49 years old (2.1 terms, SD: 1.0), and 50 to 59 years old (2.6 terms, SD: 1.4).

When comparing the age groups, there was a difference between ages 30 to 39 and 40 to 49 (*p* < 0.001), 30 to 39 and 50 to 59 (*p* < 0.001), and 40 to 49 and 50 to 59 (*p* < 0.001). We further examined the relationship between three variables: age (objective variable), FCS score (explanatory variable), and the number of extracted terms (explanatory variables). There was a significant relationship between age and the FCS score (*p* = 0.005, r^2^ = 0.255), which indicated that the FCS score increases with aging.

## 4. Discussion

Facial complexion, skin tone, and esthetic appearances have been evaluated using instruments and survey methods. There are various custom-made survey questionnaires available. A survey form should be tested for internal consistency, inter-item relationships, and reproducibility. According to our knowledge, there is no questionnaire that has been adequately tested for its reliability and validity.

In the present study, we examined the reliability and validity of FCS that could be utilized with cosmetic acupuncture treatment. Several FCS items showed reliability between items, which is consistent with our previous study [11]. However, a vast majority of the respondents could not understand some of the terms used in FCS (e.g., “infraorbital folds” 59.2% and “marionette lines” 61.3%). For practical usage, we realize that additional explanations would be required for specific terminologies used in the FCS. Furthermore, the relationship between the state of concern of the facial regions and the FCS items showed a correlation, indicating that each FCS item reflects the facial region’s state of concern. In particular, the difference between age groups and FCS score suggested that the level of concern in each FCS item increases with age. Although usage of mechanical devices could be useful to objectively evaluate the appearance of the facial skin, such as hyperpigmented spots and wrinkles, they are not capable of determining age [12]. Our FCS is a simple and practical tool to evaluate patients’ concerns regarding their skin’s appearance. Previously, we investigated the efficacy of cosmetic acupuncture using FCS [5,6,7]. Our studies demonstrated significant changes in FCS following facial cosmetic acupuncture procedures.

There are some limitations to this study. This study was based on a subjective analysis of facial appearance. We have not conducted any correlation analyses against objective parameters, such as dermal fluids, oil content, and skin surface topographic assessments. Using the FCS in conjunction with objective facial skin examinations may provide comprehensive information when evaluating the efficacy of cosmetic acupuncture treatment. In addition, it is essential to carefully monitor any potential adverse events. As cosmetic acupuncture has been gaining popularity in recent years, adverse events related to the procedure have been reported [13,14,15]. It should be noted that the vast majority of the study participants were between 30 and 50 years old. It would be useful to conduct a study among broader age groups. Lastly, the FCS used in this study was developed in Japanese. The reliability and validity of the FCS were tested among the native Japanese spoken population. The construction of other language versions is required when determining the applicability of FCS among other populations.

## 5. Conclusions

The number of extracted terms and the FCS score showed a moderate correlation. The FCS can be used as a simple and practical tool to evaluate facial appearance and assess satisfaction levels of patients who underwent cosmetic acupuncture or other facial skin rejuvenation procedures. Further studies are needed to confirm the reliability and validity of the FCS in various clinical and beauty care settings.

## Figures and Tables

**Table 1:** The 12 items on the facial check sheet.

	Not at All Concerned	Slightly Concerned	Somewhat Concerned	Moderately Concerned	Extremely Concerned
1. Periorbital lines	1	2	3	4	5
2. Glabellar frown lines	1	2	3	4	5
3. Horizontal forehead lines	1	2	3	4	5
4. Nasolabial folds	1	2	3	4	5
5. Infraorbital folds	1	2	3	4	5
6. Marionette lines	1	2	3	4	5
7. Infraorbital skin laxity	1	2	3	4	5
8. Cheek laxity	1	2	3	4	5
9. Corner of the mouth lines/laxity	1	2	3	4	5
10. Facial shape/contour	1	2	3	4	5
11. Complexion	1	2	3	4	5
12. Blotchiness and hyperpigmented spots	1	2	3	4	5

Each item was rated by the 5-point Likert scale.

**Table 2:** Extracted terms from sentences in the stated concern regarding the facial region.

No	Term	n	%
1	Blotchiness and hyperpigmented spots	67	47.2
2	Wrinkles	52	36.6
3	Skin laxity	45	31.7
4	Nasolabial folds	31	21.8
5	Corners of the eyes	16	11.3
6	Dryness	15	10.6
7	Dark circles	10	7
8	Pores	8	5.6
9	Dullness	8	5.6
10	Forehead	5	3.5
11	Resilience	5	3.5
12	Freckles	4	2.8
13	Between the eyebrows	4	2.8
14	Complexion	4	2.8
15	Breakout	3	2.1
16	Melasma	3	2.1
17	Rough skin	3	2.1
18	Corner of mouth	2	1.4
19	Mole	2	1.4
20	Eczema	2	1.4
21	Sebum (oily skin)	2	1.4
22	Swelling	2	1.4
23	Roughness	1	0.7
24	Bruising	1	0.7
25	Downy hair	1	0.7
26	Eye appeal	1	0.7
27	Darkness	1	0.7
28	There is a left–right difference	1	0.7
29	Sunburn	1	0.7
30	Instability	1	0.7
31	Big nose	1	0.7
32	Makeup paste	1	0.7
33	Facial shape/contour	1	0.7

## Data Availability

Not applicable.
